# Feasibility assessment of an occupational therapy lifestyle intervention added to multidisciplinary chronic pain treatment at a Danish pain centre: a qualitative evaluation from the perspectives of patients and clinicians

**DOI:** 10.1080/17482631.2021.1949900

**Published:** 2021-07-12

**Authors:** Svetlana Solgaard Nielsen, Jeanette Reffstrup Christensen, Jens Søndergaard, Vicki Oldenschläger Mogensen, Anette Enemark Larsen, Søren T. Skou, Charlotte Simonÿ

**Affiliations:** a Research Unit of User Perspectives and Community-based Interventions, Department of Public Health, University of Southern Denmark, Odense, Denmark; b The Research Unit PROgrez, Department of Physiotherapy and Occupational Therapy, Naestved, Slagelse & Ringsted Hospitals, Slagelse, Denmark; c Research Unit of General Practice, Department of Public Health, University of Southern Denmark, Odense, Denmark; d Master Programme for Occupational Science and Occupational Therapy, University of Southern Denmark, Odense, Denmark; e Department of Occupational Therapy, Institute of Therapy and Midwifery Studies, Faculty of Health Sciences, University College Copenhagen, Copenhagen, Denmark; f Research Unit for Musculoskeletal Function and Physiotherapy, Department of Sports Science and Clinical Biomechanics, University of Southern Denmark, Odense, Denmark

**Keywords:** Program evaluation, pain management, pain clinics, rehabilitation, health behaviour

## Abstract

**Purpose:** As part of intervention feasibility evaluation before conducting a clinical trial, this study aimed to investigate perspectives of patients and clinicians involved in the occupational therapy lifestyle-oriented programme REVEAL(OT) [Redesign your EVEveryday Activities and Lifestyle with Occupational Therapy] which was added to multidisciplinary chronic pain treatment.

**Methods:** We conducted three focus group interviews, two with eight voluntarily selected patients and one with four clinicians. Data were analysed using Braun & Clarke’s semantic data-driven analysis.

**Results:** Patients reported satisfaction with the intervention and a greater acceptance of living with chronic pain through increased understanding of pain mechanisms, more effective daily planning and improved social interaction. Patients felt empowered to change lifestyle habits by restarting habitual interests, prioritizing joyful occupations for improved occupational balance, and lifestyle modifications. Contact to occupational therapists and peer support were important empowering factors for working with lifestyle goals. Patients and clinicians expressed their views on further improvement of the REVEAL(OT).

**Conclusions:** Patients and clinicians found the lifestyle-oriented occupational therapy programme relevant as an add-on to the multidisciplinary chronic pain treatment. A need was expressed for a reduced information and treatment load and a higher degree of communication and cooperation among the clinicians involved in the intervention.

## Introduction

The worldwide weighted prevalence of chronic pain in the adult population is estimated to be about 20% (Andrew et al., 2014). Chronic pain has large negative personal and socio-economic consequences such as severe disturbances in work, domestic chores, child caring and studying, and high health care costs (Kronborg et al., 2009). Multidisciplinary biopsychosocial treatment meets chronic pain patients’ needs and is cost efficient (Kronborg et al., 2009; Scascighini et al., 2008). As a stand-alone solution, none of the available non-pharmacologic treatment modalities is superior which urges different treatment options to be included in comprehensive chronic pain rehabilitation (Turk, 2002). Non-pharmacologic treatment options and team-based approaches with a follow-up were top-ranked by patients as effective facilitators promoting their chronic pain rehabilitation (Becker et al., 2017).

Lately, the need for comprehensive programmes focusing on lifestyle in chronic pain patients has been highlighted (Nijs et al., 2020). A vicious circle of chronic pain often leads to improper lifestyle choices such as inactivity, improper nutrition and isolation, and impairs the everyday life in work, leisure and self-care. This in turn can lead to poorer mental and metabolic health and elevate risks of comorbidity with other severe health states such as heart disease, diabetes and stroke. However, many health-related lifestyle factors such as physical activity, eating habits, smoking, alcohol consumption, and stress are modifiable and eligible for inclusion in healthcare interventions (van Hecke et al., 2013). Another interpretation of lifestyle is the individual’s way of living formed through occupational engagement and performance of daily activities determined by personal needs, wishes, and resources (Velde & Fidler, 2002, p. 10). Several examples provide evidence of how professional occupational therapy assistance can promote a healthier lifestyle in people living with chronic pain by helping them perform everyday occupations that are value-based, healthy, and balanced (Clark et al., 2015; Lagueux et al., 2018, 2021; Simon & Collins, 2017).

Building on the MRC framework and supporting literature (O’Cathain et al., 2019), we developed an occupational therapy intervention REVEAL(OT) [Redesign your EVEveryday Activities and Lifestyle with Occupational Therapy] combining the following elements: a) occupational therapy evidence on lifestyle management of chronic pain; b) population-centred information on health-related quality of life, health, lifestyle, and motivation for changing lifestyle; and c) attention to how the intervention can potentially be implemented in the existing multidisciplinary treatment (usual care) for chronic pain at a Danish pain centre. The knowledge acquired throughout the intervention development process will be published in a scientific report after the final feasibility round and contain all the relevant references to collect the evidence from the research activities conducted.

According to the Medical Research Council (MRC) guidelines, complex interventions should be developed and pilot-tested before being evaluated in a clinical trial (Abraham et al., 2015). A qualitative evaluation offers insight into stakeholders’ perspectives in a healthcare intervention and may reveal facilitators and barriers for implementation to serve clinical reasoning and support treatment choices (Moore et al., 2015; Rycroft-Malone & Burton, 2015). This study aimed to evaluate user perspectives from participation in the initial feasibility study of the lifestyle-oriented occupational therapy intervention conducted as an add-on treatment to usual care to identify its benefits and challenges for participating patients and clinicians and inform the design and conduct of a future clinical trial.

## Materials and methods

### Design

Following the nature of applied research, this qualitative evaluation adopted the realist paradigm explaining reality through individual experiences of the stakeholders involved in a healthcare intervention either as recipients or deliverers. Two focus group interviews with patients and one with clinicians were conducted using semi-structured interview guides inspired by Halkier (Halkier, 2016, pp. 23–50). Data analysis was guided by the data-driven qualitative semantic approach proposed by Braun & Clarke, with systematic inductive coding and the development of patterns throughout the data (Braun & Clarke, 2006).

### Setting

This study was conducted from April to October 2019 in cooperation between Naestved, Slagelse and Ringsted Hospitals (Region Zealand, Denmark) represented by the department for physiotherapy and occupational therapy and its occupational therapy unit (OTU, Naestved Hospital), the department of anaesthesiology and its multidisciplinary pain centre (MPC, Naestved Hospital), and the University of Southern Denmark. The MPC has been using the bio-psychosocial model for chronic pain treatment since 2014 and delivered chronic pain management based on cognitive-behavioural therapy (CBT) led by a multidisciplinary team of physicians, nurses, physical therapists, psychologists and a social worker. The cooperation between the OTU and the MPC aimed to include the REVEAL(OT) into the multidisciplinary treatment practice.

### Intervention

**Figure 1.**
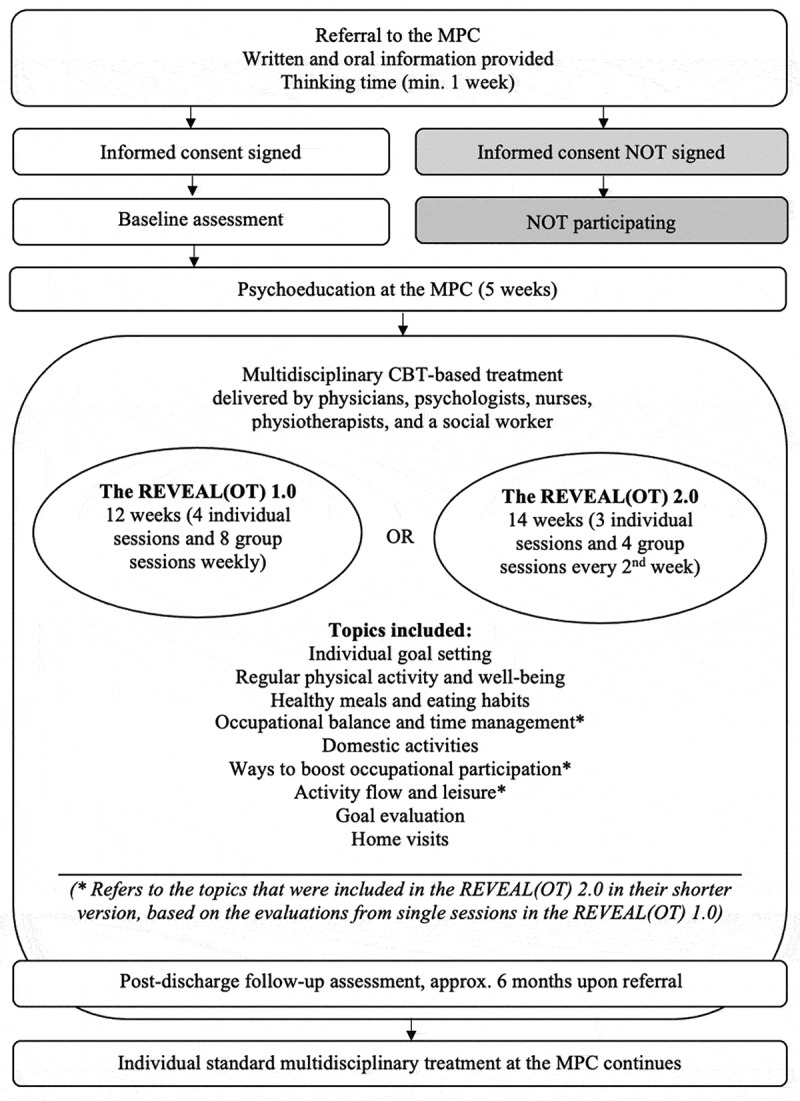
The structure of the REVEAL(OT) (versions 1.0 and 2.0)

The lifestyle-oriented REVEAL(OT) intervention (Clinicaltrials.gov reg. NCT03903900; Region Zealand, Journal Number: SJ-703) underwent a feasibility evaluation. Usual care at the MPC started with a compulsory 5-week preparatory psycho-education course where all the healthcare disciplines represented their field of impact for the patients followed by an individually tailored treatment course. The REVEAL(OT) ran in parallel with usual care. Two versions of the REVEAL(OT) (1.0 and 2.0) (Figure 1). were subject to the feasibility evaluation. REVEAL(OT) 2.0 was an improved intervention based on the experiences from REVEAL(OT) 1.0, with reduced treatment intensity, adjusted according to the patients and clinicians’ feedback, which should secure more proper compatibility with usual care. The REVEAL(OT) 1.0 lasted 12 weeks and contained eight group sessions of two hours and four individual one-hour sessions, providing weekly contact with occupational therapists. The REVEAL(OT) 2.0 lasted 14 weeks and contained four two-hour group sessions and three individual one-hour sessions every second week, where contact with occupational therapists also was provided.

Max. six patients were admitted pr. group. At baseline, the patients identified their occupational problems related to productivity, self-care and leisure activities that inspired further goal setting. Group sessions included information and discussion on meaningful occupations, healthy eating and daily physical activity concerning chronic pain. The patients learned about obstacles in performing human occupations and low-grade inflammation mechanisms related to improper nutritional choices and sedentary lifestyle and how human habits emerge and can be modified. The patients reflected on the importance of meaningful occupational performance for health and well-being, implementing anti-inflammatory eating principles, and tailoring regular physical activity to their everyday life. The occupational therapist leading the course provided individually tailored motivational support to promote transfer of the new knowledge and experiences to the patients’ everyday life. Skill training in performing challenging activities of daily living and lifestyle diaries for monitoring personal lifestyle goals related to occupational performance, healthy eating, and physical activity were implemented to help the patients smoothly transform the new knowledge to their everyday life and home environment. Individual sessions, including one or two home visits, were included to support the occupational therapy treatment tailored to the individual’s needs. Also, the patients could borrow and try a variety of assistive devices such as ergonomic chairs, seats and lumbar cushions, swivel pads, kitchen utensils, bath benches, bath brushes with ergonomic handles, sliding layers, etc. The REVEAL(OT) was protocolized and manualised to enhance fidelity among the interventionists. Cooperation between the OTU and MPC should secure coordinated planning to cover the patients’ treatment needs. Upon intervention discharge, the patients continued with their planned regular treatment at the MPC.

### Participants

In the following, we use “patients” or “clinicians” for group specification purposes and “participants” when we refer to both groups.

***The patients:*** An entire study cohort of 20 patients included in the feasibility studies was invited to participate. The inclusion and exclusion criteria for the patients followed the main study protocol (Registration number NCT03903900, Clinicaltrials.gov). Four patients took part in each of the two patient focus group interviews (FG1) and (FG2), i.e., eight patients in total. The patients were between 18 and 65 years old and had chronic pain diagnosed for at least three months. The patients harboured no acute pain or current comorbidities such as headache/migraine, cancer, depression, substance misuse, or severe psychiatric diagnoses such as psychoses. All the patients had sufficient Danish speaking skills. In focus groups 1 and 2, there were some patients who participated in version 1.0 of the REVEAL(OT), and some in version 2.0. Due to patients’ timing preferences, we could not dedicate one focus group interview to an intervention version each. We observed no markable differences in patient experience from one intervention version to the other.

Initially, 15 patients consented to participation. Later, one patient withdrew because she entered another inpatient treatment programme and was full-time occupied. Three patients were sick on the day of the interviews, and another three did not show up for unknown reasons.

***The clinicians:*** The clinician focus group (FG3) included one occupational therapist from the OTU who delivered the REVEAL(OT)-intervention, and three representatives from the MPC involved in usual care, e.g., one physician, one psychologist, and one physiotherapist. The rest of the employees at the MPC could not participate due to the current workload at the clinical units involved in usual care and the add-on intervention. Presentation of the participants—see Table I.

**Table I.** Presentation of the participants characteristics

	Focus group interview 1 (FG1)					Focus group interview 2 (FG2)					
Patients	1 (FG1P1)	2 (FG1P2)	3 (FG1P3)		4 (FG1P4)	5 (FG2P5)		6 (FG2P6)		7 (FG2P7)	8 (FG2P8)
Gender	Female	Female	Female		Male	Male		Female		Female	Female
Age in years	60	52	24		51	60		60		58	62
Working status	Sick leave	Home-staying	Sick leave		Disability pension	Sick leave		Working 20 hours/ week		Disability pension	Sick leave
Pain origin	Lumbar disc herniation	Fibromyalga; Primary knee osteoarthri-tis; Occupa-tional injury (back)	Lumbar disc herniation; Abdominal pain; Head-ache		Occupational injury (back)	Cervical disc herniation; Osteochond-rosis; Spinal stenosis		Primary osteoarthri-tis; Sequelae after knee surgery		Spondylolis-thesis; Poly-neuropathy; Sequelae after spinal cord herniation surgery	Hip arthritis
Social status	Living with a partner, grown-up children	Living alone, grown-up children	Living with a partner, no children		Family with children (teens)	Living alone, grown-up children		Living with a partner, grown-up children		Living with a partner, grown-up children	Living alone, grown-up children
Occupational problems^a^	Participating in social events; Domestic chores; Having a job; Gardening	Initiating and completing activities; Eating regularly; Cleaning; Cooking; Baking	Memorizing new information; Moving around (without a wheel chair)		Stacking firewood, Consuming less sweetened beverages; Increasing physical activity;	Getting ind and out of the car; Having hobby (stuffing animals); Hunting; Doing woodwork;		Cycling (on a lady bicycle); Riding; Having guests; Carrying laundry		Putting clothes on; Gardening; Washing floors; Participating in bingo games	Standing up while cooking; Performing sitting activities (pc, knitting, etc.);
(Occupational problems^a^)					Performing fine motor work; Keeping balance when shifting position	Sitting in the car					Performing dog training (as tutor); Sitting in the car; Walking by the beach (on uneven surface)
REVEAL(OT) version	1.0	2.0	1.0		1.0	2.0		2.0		2.0	2.0
	**Focus group interview 3 (FG3)**										
Clinicians	1 (FG3C1)			2 (FG3C2)			3 (FG3C3)		4 (FG3C4)		
Profession	Physician			Psychologist			Physiotherapist		Occupational therapist		
Gender	Female			Male			Female		Female		

^a^*Problems in occupational performance and participation (max. 5) identified and prioritized according to their self-perceived meaningfulness by the patients using the Canadian Occupational Performance Measure as an assessment tool (Ref. Law, M., Baptiste, S., Carswell, A., McColl, M.A., Polatajko, H., & Pollock, N. (2019). Canadian Occupational Performance Measure (5th ed.-revised). Altona, Canada: COPM Inc.)*

### Data generation

An extern researcher, who neither planned nor delivered the intervention, conducted all the focus group interviews as a moderator (VOM) to reduce conflict of interests. The researcher who planned the intervention (SSN) performed observations during all the focus group interviews.

VOM and SSN led the three focus group interviews. We strived to establish an open and trustful atmosphere to encourage participants to share their opinion of how they perceived the REVEAL(OT) programme. Two semi-structured interview guides supported the conduct of the focus group interviews with patients and clinicians (Halkier, 2016, pp. 51–70). To facilitate free discussion, both semi-structured guides included few questions with a broad focus on the subject of our interest which should invite the participants to reflect without feeling restricted to a certain kind of response. The moderator supported a balanced degree of participation using additional questions of relevance pre-determined in the interview guides. We welcomed the participants to share any relevant information, including criticism and negative or missing experiences. Moreover, we prompted the clinicians who consented to participation to collect any relevant comments regarding the REVEAL(OT) among their colleagues who were occupied with work and could not participate. We provided the participants with ergonomic sitting chairs, food and beverages during the focus group interviews. We added 15 minutes to the estimated time consumption to avoid a time rush.

First, we conducted two focus group interviews with the patients. We asked the patient groups: 1. How did you work on changing your everyday habits during the REVEAL(OT) intervention?; 2. How did you implement experiences from the REVEAL(OT) intervention to everyday life?; 3. Please elaborate on the benefits and challenges of the programme; 4. How would you describe the most prominent effects of the REVEAL(OT) intervention on your daily life?

**Figure 2.**
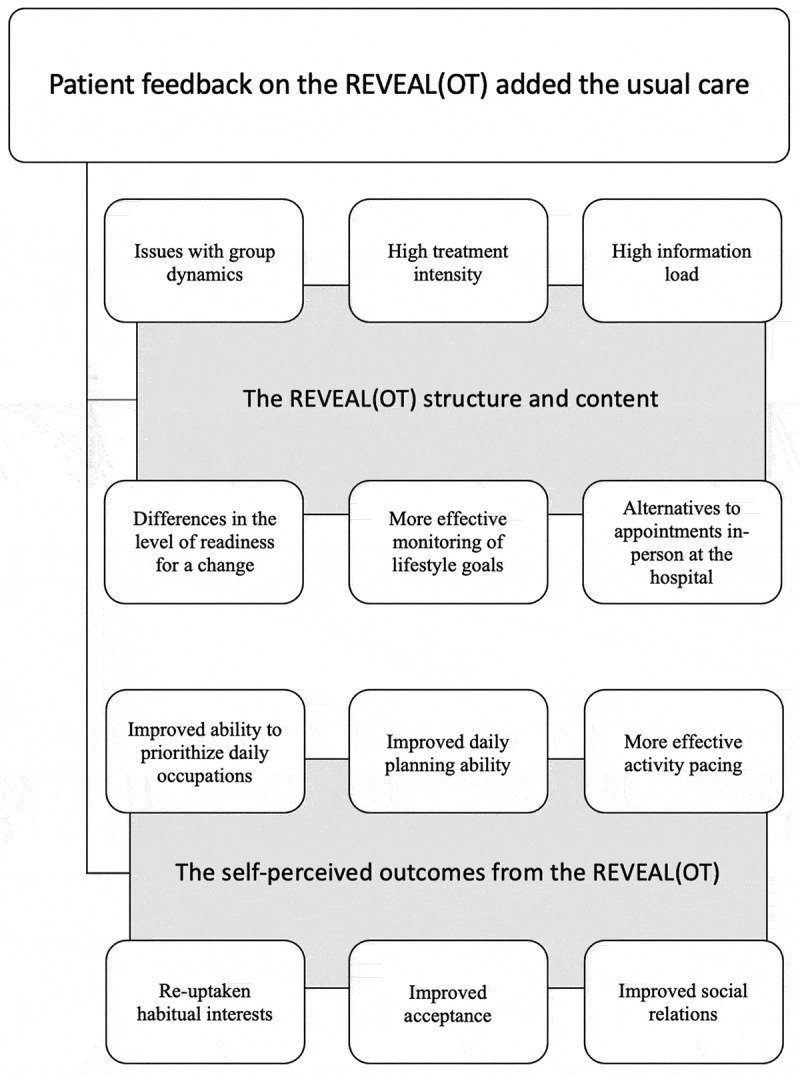
Selected patient statements as a moderation tool for interviewing the clinicians

Second, we interviewed the clinicians, starting with a visual presentation of selected statements from the focus group interviews with the patients (Figure 2). We considered all the clinicians as one multidisciplinary clinical unit involved in the feasibility testing process regardless of their formal affiliation with either OTU or MPC. We asked the clinicians: 1. How would you describe your role and work concerning the REVEAL(OT) intervention as a novel treatment option added to usual care?; 2. Please reflect on the selected patients’ statements and propose a further improvement of the REVEAL(OT) as an add-on treatment option incorporated into usual care; 3. Please evaluate the REVEAL(OT) regarding its strengths and limitations. 4. Please identify which outcomes the multidisciplinary chronic pain treatment should target to be effective.

The focus group interviews lasted 90 to 120 minutes. The interviews were recorded and transcribed verbatim by an extern assistant not affiliated with the current research.

### Data analysis and interpretation

Seventy-six pages of transcribed single-line raw text were collected. The semantic analysis followed the six phases proposed by Braun & Clarke (Braun & Clarke, 2006). In Phase 1, SSN and VOM became familiarized with the data, comprehending the entire transcribed text, and obtained an overall understanding of the data. In Phase 2, the two authors generated initial codes independently using NVivo 12 software, version 12.6.0, and discussed discrepancies. The discrepancies emerged from a variety of possible interpretations when coding the text. Interactions with spouses could be coded as “Spousal involvement”, “Partner support”, or “Family involvement” if children were included. We decided to allow a more general description for social relations because formal details were less important. For example, “Partner” was preferred over “Husband” or “Spouse”. At the same time, we decided to focus on the contribution of an interaction type to an individual living with chronic pain because the output of the chronic pain treatment was in both patients and clinicians’ concerns. Thus, words “Support” or “Accept” was preferred over those of more neutral character such as “Involvement”. In Phase 3–5, searching, reviewing, defining and naming the themes that emerged from the initial codes were performed by SSN and CS. We needed a similar approach when reviewing and defining codes both from the patients and clinicians’ perspectives. Thus, we decided to focus on the contributive role of each code in chronic pain treatment to guide us in further code grouping. As in the case of social interactions, we used distinguished codes defining phenomena that emerged within an individual living with chronic pain such as “Feeling accepted by others”, from those of an external character such as “Supportive environments”. In Phase 6, all authors contributed to the understanding and discussing the findings before elaborating on a cumulative report in the results and discussion sections. We included selected citations from the interviews to add transparency to the results. Relevant research supporting the discussion was applied to place the findings in a broader context and reveal commonalities and discrepancies.

### Ethical considerations

The Ethical Committee in Region Zealand (SJ-703) and the Danish Data Protection Committee in Region Zealand (REG-052-2018) approved the study. We distributed an invitation with detailed information on the investigation to the potential participants at least one month before asking them to sign informed consent. All the participants were informed about the focus group method, who the researchers were and how they would perform the investigation. We underscored that the focus group interviews would be subject to the confidentiality of personal data. The information provided before the investigation was repeated orally before starting each focus group interview. The first author provided oral and written information about the study and obtained consent at least one month before the first focus group interview. The participants received information on voluntary participation (i.e., both participation in the investigation itself or its parts, inclusive the right not to answer a question if not feeling comfortable), unrestricted withdrawal, confidentiality, anonymizing, and archiving the data. For data recording, storage and sharing, we used voice recorders, encrypted USBs, and software, approved for research activities following the General Data Protection Regulation (Regulation (EU) 2016/679). No adverse events caused by participation in the study were expected to occur.

## Results

The eight patients and four clinicians who participated in the three focus group interviews (FG1-3) are presented in Table I.

The iterative reading of the transcribed text of FG1 and FG2 revealed that after participation in REVEAL(OT), the patients felt well-supported in managing and living with chronic pain in new and valuable ways. In addition, the patients provided recommendations for further enhancement of the intervention. Data from FG3 with the clinicians supported that the REVEAL(OT) was beneficial as an add-on to usual care and pointed out the necessity to adjust the information load, treatment intensity and multidisciplinary cooperation. Furthermore, the clinicians added suggestions for organizational amendments to improve the REVEAL(OT) implementation along with the existing pain management concept. The cumulative analysis included two themes: “Increased patient acceptance of living with chronic pain” and “Empowering patients to make lifestyle changes”.

### Increased patient acceptance of living with chronic pain

The patients expressed that their primary expectation from the chronic pain treatment at the MPC and OTU was pain reduction. When they realized that this was not realistic, the aim became to improve coping with pain in everyday life. As the most prominent output from the REVEAL(OT), the patients highlighted an increased acceptance of living with pain. They described that their acceptance grew because of greater awareness of their own needs and wishes and the acquired new ways of managing daily living with chronic pain. They could now skip the role of always sacrificing themself for others. They became more skilled in structuring their everyday activities, as described by one of the patients:

Well, all those small domestic chores, they are running up all the time. I say “small” because I was doing those all the time. (…) [Now] I learned to give the others small tasks, acting smoothly instead of being tough and just doing it myself. One day they [children] were cooking, filling in the dishwasher and washing clothes. This way, I can concentrate on what I have to. I can now do the things I enjoy (FG1P4).

The patients experienced that their energy level and personal demands had changed after chronic pain onset. Guided by occupational therapists, they learned how to incorporate those changes in their everyday life. Accordingly, they found it easier to ask for help:

“A change happened about cooking. I’m better now to ask if he [the patients’ husband] would peel potatoes. So we are sharing workload” (FG2P8); “I’m aware now that there are other ways to handle things. I appreciate it deeply” (FG2P5); “I look at myself with new eyes, so I think that is the most important output of being here” (FG2P5).

The increased acceptance of living with chronic pain became particularly visible when the patients faced their social environment while they gradually became able to cease suppressing their own needs and fear of making others disappointed. The patients emphasized that awareness of their standpoint helped remove barriers between themselves and their social environment. One of the patients summarized it, while the others mutually consented by nodding their heads:

The most important thing for me was to find space for myself … and say ‘no’ to what I can’t or don’t want or have the capacity for (…) That it is okay not to be able to cope with all that I was used to before. It has helped me, indeed (FG2P8).

The thoughts echoed in the patients’ focus group that brought examples on how to stop being “a pleaser” and become more selective when inviting guests. A new understanding of how to set limits was valued as a personal strength and no longer seen as unpoliteness. Comprehending the patients’ thoughts, one of the clinicians perceived that the patients became more self-assertive and explicit: *“When they begin to communicate in a new way and express their needs in terms of pain, then they show more respect for their pain condition and get more skilled in setting clear limits. One’s social environment can handle that much more easily!”* (FG3C4).

The patients outlined that they also became better at being included in social groups while following the REVEAL(OT):

Meeting other people that understand one’s situation can also enhance the quality of life. I felt myself being all alone. That is why it is an incredible feeling to come here and be taken seriously, with that solution-oriented approach to things, pursuing the best possible result for you as a patient (FG2P5).

The feelings of acceptance and being accepted are complex mechanism because, as one of the clinicians put it: *“We cannot force anyone to accept one’s condition. This is a process, and we do not know how long it would take. It is definitely a challenge for every clinician”* (FG3C4). From the clinicians’ view, acceptance was linked to greater understanding and coping with pain.

The clinicians noted that close social network—especially spouses—may find it extremely difficult being caregivers for a person with chronic pain. Some of the patients expressed that they wished the REVEAL(OT) had informed their spouses about the intervention and how to support them:

People expect one to get better after coming here. It was difficult to explain what we were doing here. You meet two hours a week, then eat more vegetables, drink water, and get more physically active … But what else was it for? It would be fine for the nearest ones to get some more information, just briefly (FG1P3).

### Empowering patients to make lifestyle changes

The enhanced understanding of processes behind chronic pain highlighted by the clinicians in the previous theme was confirmed by the patients to be a starting point for them in changing habits. A patient said: *“It has been fantastic to understand that it is okay to peel some potatoes and then relax and sit and knit, and then to return and make some meatballs or whatever. (…) Because you manage those pain flares better”* (FG2P8).

The patients valued empathic professional communication as an empowering factor. It meant a lot for them that the occupational therapists were “*accommodating and friendly, and wished you*[patients] *the best”* (FG1P1). They described that active listening and involvement in one’s personal life situation boosted their self-esteem and encouraged them to try out new things. The patients preferred face-to-face contact with clinicians. Alternatively, a communication platform allowing visual decoding (video instead of phone calls), because it worked empowering: *“I need to … to be able to ‘read’ people when I talk to them”* (FG1P1). However, phone calls were still considered applicable, but rather in the later sessions, in cases where contact already had been established.

Another empowering factor for patients in making healthier lifestyle choices was peer support during the REVEAL(OT). Peers inspired the patients to work with personal goals, maintain a healthy lifestyle and broaden perspectives: *“It helps to talk with others in the same situation because one tends to feel being the only one in the whole world who has it like this”* (FG1P3); *“It helps to support each other in getting new experiences”* (FG2P6).

The patients found peer support in the REVEAL(OT) beneficial for their treatment but also challenging. Most patients considered a well-functioning peer collaboration to be associated with readiness to share useful personal experiences and being ready to try new things: *“I think participating in such an intervention, one must have to keep the mind open and be receptive”* (FG1P3). At the same time, some patients were sensitive to (self-perceived) lack of engagement in peers, as it made them feel demotivated and could provoke conflicts within a group, demanding early conflict management.

In some patients, changing lifestyle began with restarting habitual interests, previously paused due to chronic pain: *”I started to knit again, both on a knitting machine and manually. But then I thought, I could also make some patchwork and dog collars as I used to but didn’t have surplus to during the past two years”* (FG2P8). Though the activities did not ease the pain but could cause more pain afterwards, the patients did not regret their choice because of earning extra energy and joy from doing the value-based occupations: *“I know*, [participating in a choir concert] *will be pretty tough. I will be feeling in chatters the whole next week. But this is what I want”* (FG1P2); *”I have been hunting again! For the first time in the past four years, I had a surplus for that, despite the pain”* (FG2P5).

During the REVEAL(OT) programme, several strategies helped the patients revise and rearrange their daily structure to create space for pleasurable events, which implied a more balanced everyday life. Making value-based choices and activity pacing were the methods widely used for saving and gaining energy:

“I split the tasks because I can’t do that much cleaning. (…) I have also learned to structure my day so that I both get some rest, do exercises, and walk for half an hour every day” (FG1P1); “During the past month, I left some arrangements earlier because I also had plans for the next day and I also want to be able to do things tomorrow” (FG1P2); “I never make plans two days in a row. Always one day with plans, then a break for one day or maybe two” (FG1P3).

Besides doing more meaningful occupations, the REVEAL(OT) empowered the patients to live a healthier lifestyle. Using lifestyle diaries and regular follow-ups prompted sustainable habit changes. Satisfaction with the intervention appeared high in those who successfully achieved their lifestyle goals: *“I feel like I just can’t live without that water. It makes a difference!*” (FG1P2); *“I have eaten more fruit and vegetables. I haven’t been good at it before”* (FG1P1).

Lifestyle diaries were considered an empowering tool for adaptation and maintaining lifestyle changes because knowledge and the initial motivation did not automatically lead to changing lifestyle habits. The element of practising in real life had a crucial influence on achieving personal lifestyle goals.

Using assistive devices during the REVEAL(OT) made everyday chores “doable” and was another factor considered empowering for changing lifestyle. The patients valued the opportunity highly *“to loan and try different things”* (FG1P1) while searching for a solution that matched their requirements: *”Instead of buying things* [to test them], *you knew when you bought* [an assistive device] *that you have tested it, and it worked”* (FG1P2). The latter patient have tried several lumbar cushion models and found one that enabled her to travel by car and participate in family gatherings and eating together without collapsing because of back pain.

The patients described as essential the connection between the treatment delivered in the hospital facilities and their home environment. Several patients expressed that their families would benefit from some brief information on the intervention contents. Even so, home visits met a degree of resistance in some patients who expressed that the output of the home visit was low, or the aim was not clear enough to be considered empowering for changing habits.

The parallel delivery of usual care and the REVEAL(OT) implied another barrier for empowering the patients as it reduced their overall surplus. Despite improvements made in the REVEAL(OT) 2.0 such as reduction of the information scope (fewer topics on the agenda) and the treatment intensity (from meetings every week to every second week), the patients still experienced the information and treatment load in the combined intervention to be too high. The clinicians agreed that the information and treatment load was sometimes difficult for the patients to handle. Both the patients and the clinicians proposed that the REVEAL(OT) should be carried out after the compulsory 5-week preparatory psycho-education course, running parallel with the individually tailored treatment at the MPC that possessed higher flexibility and, thus, was more compatible with the add-on intervention.

The interview with the clinicians revealed a complication in the cooperation between the MPC and OTU, as there was a physical distance between the two hospital units. Not being able to get involved in everyday clinical practice on a daily basis was considered a barrier to interdisciplinary cooperation. When patient-related questions had to wait before getting clarified, this sometimes caused confusion among both the clinicians and the patients. The clinicians at the MPC expressed a need for greater insight into the treatment elements revealed within the REVEAL(OT) and more intensive communication between the OTU and the MPC.

## Discussion

Patients’ and clinicians’ focus group-based evaluation of the REVEAL(OT) reflected patients’ increased acceptance of living with chronic pain along with patient empowerment for changing lifestyle. The participation in the REVEAL(OT) was deemed satisfactory by the patients. However, specific improvements in the intervention were needed before conducting a clinical trial.

During this investigation, we gained an insight into the patient perspective of living with chronic pain and the compromising effect of chronic pain on multiple life areas and the identity in an individual (Vlaeyen et al., 2016). We were excited to observe how even a modest tailored adjustment in occupational performance can make the mechanism of change work and became a turning point towards improved quality of life. That suggests that occupational therapy could benefit health and well-being in chronic pain (Lagueux et al., 2018), also applied to this particular clinical setting.

Occupational science, i.e., a discipline systematically studying human occupations, participation and their relations to human health, supporting occupational therapy practice, links occupational engagement with our identity and the roles we perform throughout our lives, and their crucial importance for human health (Christiansen, 2004; Hocking, 2013). Benefits of improved occupational performance for health and well-being in people living with chronic pain have been seen in other occupational therapy research (Lagueux et al., 2021; Simon & Collins, 2017). The REVEAL(OT), like other nonpharmacological treatments of chronic pain, aims to enhance human coping ability through greater awareness of their own needs and wishes and less social avoidance (Turk & McCarberg, 2005). The unique role of the REVEAL(OT) as an add-on to usual care at the MPC could be attributed to its impact on the occupational dimensions of doing, being, becoming and belonging, essential for health and well-being (Wilcock, 1998; Yazdani & Bonsaksen, 2017). In the dimension of doing, patients participating in the REVEAL(OT) discovered new ways to perform meaningful or purposeful activities, experimented with new healthy recipes, and reduce sedentary time. In the dimension of being, patients established new healthy routines determined by their value-based choices. In the dimension of becoming, the focus shift from chronic pain diagnosis and disability to joyful or purposeful everyday content and experienced themselves as actively making a meaningful difference in their lives. Meeting others and sharing experiences made patients feel belonging to an active group of people capable of coping with everyday obstacles and breaking out of the vicious circle of chronic pain.

Moreover, the REVEAL(OT) would bring a lifestyle-oriented focus into the existing chronic pain management, which international studies have previously proposed (Lagueux et al., 2018; van Hecke et al., 2013). Particularly, interventions targeting multiple (≥ 2) lifestyle factors within the same intervention has been urged (Nijs et al., 2020). The REVEAL(OT) explored how several relevant lifestyle factors can be tackled within chronic pain treatment at a Danish outpatient clinic, assisted by occupational therapists.

Specific clinical practice-bound differences between usual care and the lifestyle-oriented occupational therapy intervention were apparent. However, we have not found the two treatments to be conceptually opposed. The effect of CBT, on which the MPC has based its treatment, is evident (Skelly et al., 2020). While CBT focuses on cognitive processes such as thoughts, emotions, bodily sensations and behaviour, the REVEAL(OT) primarily worked with the behavioural dimension linked to performing meaningful everyday activities. The REVEAL(OT) targeted occupational behaviour concerning occupational problems identified at the intervention entry. Occupational therapy assessments applied in the REVEAL(OT) could deliver information helpful to the other healthcare disciplines at the MPC such as weekly activity schedules or clinical reports on working with occupational goals. If occupational therapy became an integrated part of usual care, occupational therapists could assist in measuring and evaluating the treatment effect on occupational performance and satisfaction, which some evidence links to self-efficacy (Thomas et al., 2020). Self-efficacy is, in turn, associated with chronic pain prognosis (Martinez-Calderon et al., 2018). The Danish private residential rehabilitation clinics use occupational therapy and a lifestyle-oriented approach in multidisciplinary chronic pain treatment (Schmidt et al., 2018). However, it is unknown how to apply their experiences to a public outpatient chronic pain clinic context, indicating the need for further investigation.

Further improvements in the REVEAL(OT) were needed, of which the reduction of the information and treatment load was the most crucial for the patients. Several solutions for reducing treatment and information load in the REVEAL(OT) could be considered. Firstly, the handouts for group sessions could be printed as a patient handbook to free the patients from systematizing and archiving the handouts by own hand. That would also facilitate patients’ overview of the intervention contents and prevent possible loss of relevant materials. Secondly, conducting the REVEAL(OT) after the compulsory psycho-education course, i.e., in parallel with the individual consultations at the MPC, could also release more surplus in the patients. Decreasing cognitive demands during the intervention would add energy surplus to the patients which is necessary to accommodate lifestyle changes (Nijs et al., 2020). Lower cognitive load also positively influences the group dynamics by releasing working memory, which is beneficial for communication skills such as the ability to take others’ perspectives (Cane et al., 2017). At last, some programme elements such as home visits could be made optional when found relevant in cooperation with a patient. Though some patients would wish their close network received a brief information letter on the REVEAL(OT) contents and its impact, healthcare personnel entering home environments was seemingly perceived intimidating to some degree. Involving patients in decision making about their treatment and reflecting patient’s actual needs are essential qualities of adequate pain rehabilitation (Oosterhof et al., 2014).

To improve the multidisciplinary cooperation, the clinicians proposed more frequent meetings between the clinical units involved and making the occupational therapy contribution to usual care more explicit and valuable. The benefits of having clinical units placed close to each other underpinned by the clinicians as an essential factor that would have improved multidisciplinary cooperation was eye-opening for us and inspired us for seeking new solutions. The Danish Health Authority has recently highlighted the role of occupational therapy in chronic pain rehabilitation and urged the inclusion of the occupational therapy competencies in multidisciplinary chronic pain management as its necessary treatment option, yet poorly represented in this area of healthcare in today Denmark (The Danish Health Authority, 2020). The REVEAL(OT) represents the first Danish experience of how occupational therapists can promote occupational performance and participation in chronic pain patients referred to a public Danish pain management centre. Findings from this study will inform the next feasibility phase in developing the REVEAL(OT) programme and inform the design and conduct of a future clinical trial. We hope that the REVEAL(OT) if found effective, will inspire stakeholders for including occupational therapy in the multidisciplinary treatment of chronic pain.

## Limitations

This study’s findings must be interpreted with the premises of qualitative focus group interview studies in mind. Though the small study sample has only provided an insight into opinions and experiences of this particular group of chronic pain patients and clinicians, the participants represented forty per cent of the potential cohort of patients and clinicians who may have an opinion about, or experience with, the add-on intervention, which could be considered representative for this specific patient cohort (Vasileiou et al., 2018). A higher participation rate of both patients and clinicians might have revealed different themes and other interpretations relevant to the intervention evaluation. However, reflecting the two themes that emerged from the data analysis in this study, we believe that our findings may speak into similar experiences in a broader range of people living with chronic pain and multidisciplinary healthcare workers.

The patients who took part in the focus group interviews were probably more resourceful and motivated than those who refused to participate. Additional relevant data on the participants could have prompted further analysis, which remained unexplored in this study because of the few data categories included. Additionally, an apparent limitation was the non-participating nurses in the sample, though the representation of this group of healthcare professionals was the largest in the clinical setting studied. Both factors mentioned above elevated the risks of sampling bias in this study (Cheung et al., 2017). Involvement of all the healthcare disciplines represented within the multidisciplinary chronic pain treatment and its add-on treatment option, as well as a broad representation of patients with lower capacity for participation, would have provided us with perspectives from a broader scope of impactful stakeholders in the intervention development process (Concannon et al., 2014).

The semantic data-driven analysis claims that there is not only one way to identify themes in a dataset (Braun & Clarke, 2006). However, all the co-authors made efforts to secure that the analysis and interpretation of the results in this study would adequately reflect the opinions and experiences of patients and clinicians.

## Conclusions

The patients were satisfied with the lifestyle-oriented occupational therapy programme REVEAL(OT) that promoted increased patient acceptance of living with chronic pain and empowered them for changing lifestyle. The patients and clinicians considered the REVEAL(OT) a relevant add-on to usual care and proposed further improvements such as reducing the information and treatment load and a higher degree of professional communication and cooperation.

## Data Availability

Data supporting the results of this study can be accessed by contacting the corresponding author. https://portal.findresearcher.sdu.dk/en/persons/ssolgaard.
